# A rare case of persistent air leak: beware of all the tubes

**DOI:** 10.1186/s12245-021-00381-6

**Published:** 2021-09-22

**Authors:** Amarja Ashok Havaldar, Abdul Rahim Fazal, Abhilash Chennabasappa

**Affiliations:** Department of Critical Care, St. John’s Medical College, Bangalore, 560034 India

**Keywords:** Air leak, Mechanical Ventilation, Neuromuscular weakness, Respiratory failure

## Abstract

**Background:**

Patients with acute respiratory failure, impaired consciousness, and impaired airway reflexes will require invasive mechanical ventilation. Monitoring of such patients is important. The use of ventilator scalars and loops help in monitoring, diagnosing the abnormality, and treating the patients effectively. We report a rare cause one should suspect in a case of persistent and fixed air leak in a patient requiring mechanical ventilation.

**Case presentation:**

We describe a 28-year-old young patient requiring ventilator support due to neuromuscular weakness. His neuromuscular weakness was rapidly progressing involving the respiratory muscles. The patient was intubated and put on mechanical ventilator support. He was transferred from another health care center to our hospital. On evaluation, the patient was intubated with ETT no 8. The patient had persistent air leak as observed on the ventilator graphics. We checked for ETT cuff malfunction, ventilator circuit, catheter mount, and HME for any disconnection causing the leak. The air leak which we observed in our patient was due to the malpositioned Ryle’s tube.

**Conclusions:**

Vigilant monitoring of patients requiring mechanical ventilation is necessary. For the evaluation of the cause of air leak, algorithmic approach will help in correctly identifying the abnormality.

## Background

Monitoring of patients requiring mechanical ventilation is important. The use of Ventilator scalars and loops help in monitoring, diagnosing the abnormality, and treating the patients effectively. The use of Scalars helps in identifying abnormalities of resistance and compliance, presence of secretions, autopeep, and air leak. Similar changes will be observed in loops depending on the abnormality.

Presence of air leak is one of the common abnormalities one can come across. Possible causes of air leak could be ETT (Endotracheal tube) cuff leak, ventilator circuit traps which are loose, or ETT, which is of small size than the glottis. These abnormalities can be easily identified and can be corrected.

We report a rare case of persistent air leak in a 28-year-old patient (IEC ref no 006/2020).

## Case presentation

A 28-year-old young male presented with the history of weakness in both lower limbs after viral illness. Neurological assessment showed weakness of both lower limbs which gradually progressed to involve the upper limbs. The possibility of ascending flaccid paralysis was considered. He received 3 cycles of plasmapheresis. Subsequently, the patient developed breathing difficulty and required ventilator support. He was transferred to our hospital. On evaluation, the patient had ET tube and Ryle’s tube and was connected to ventilator support.

**Fig. 1:**
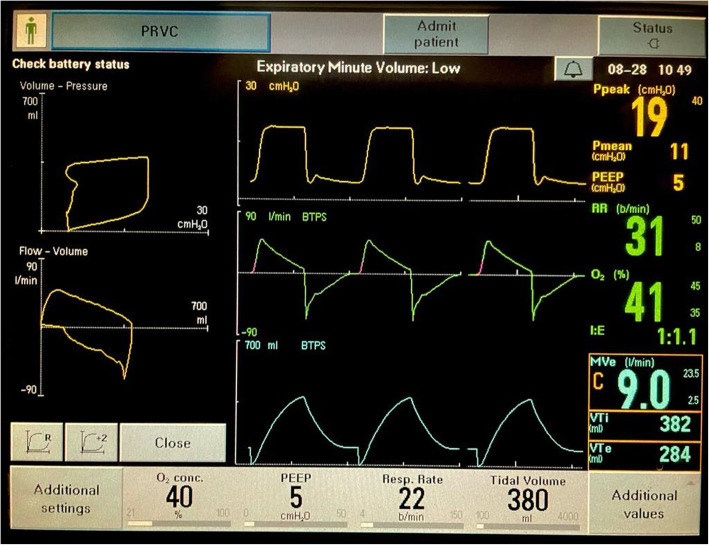
PRVC mode with air leak

Ventilator settings were as follows: PRVC mode Fio2 40%, RR 22, and TV 380 ml. On the ventilator graphics, air leak was noted (Fig. 1). ET tube position and cuff was checked. It was observed that the ET tube, no. 8, was fixed at around 18 cm. The clinical decision of check laryngoscopy after sedation and paralysis was made. On laryngoscopy, the ET tube cuff was above the glottis, and after deflation of cuff, it was difficult to advance the tube. So, the tube was changed to 8.5 no ETT and fixed at 22 cm H_2_O. Tube position was confirmed by auscultation and subsequently by ETCo_2_.

After the change of ET tube, the leak persisted. So, the ventilator circuit and water traps were checked for any air leak. There was no subcutaneous emphysema, and lung ultrasound showed good lung sliding. So, we planned on doing CXR.

**Fig. 2:**
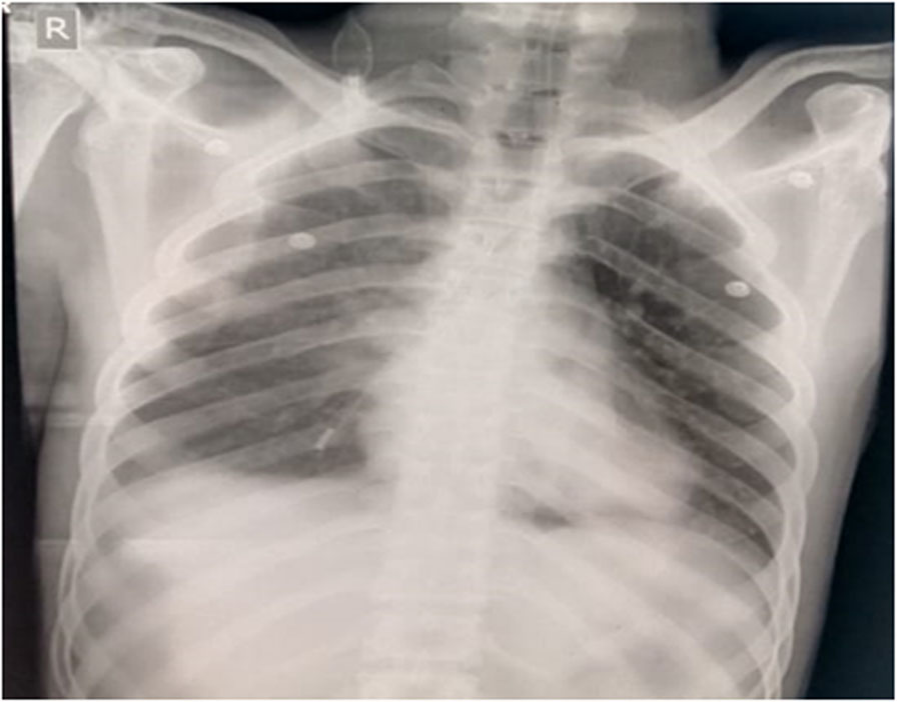
CXR showing malpositioned Ryle’s tube

**Fig. 3:**
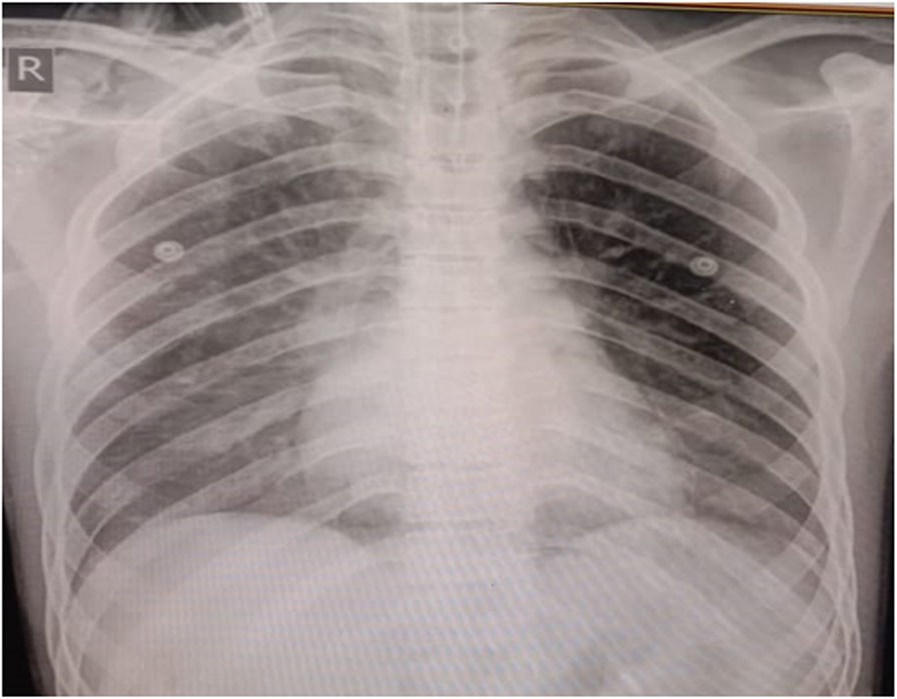
CXR after correct placement of Ryle’s tube

**Fig. 4:**
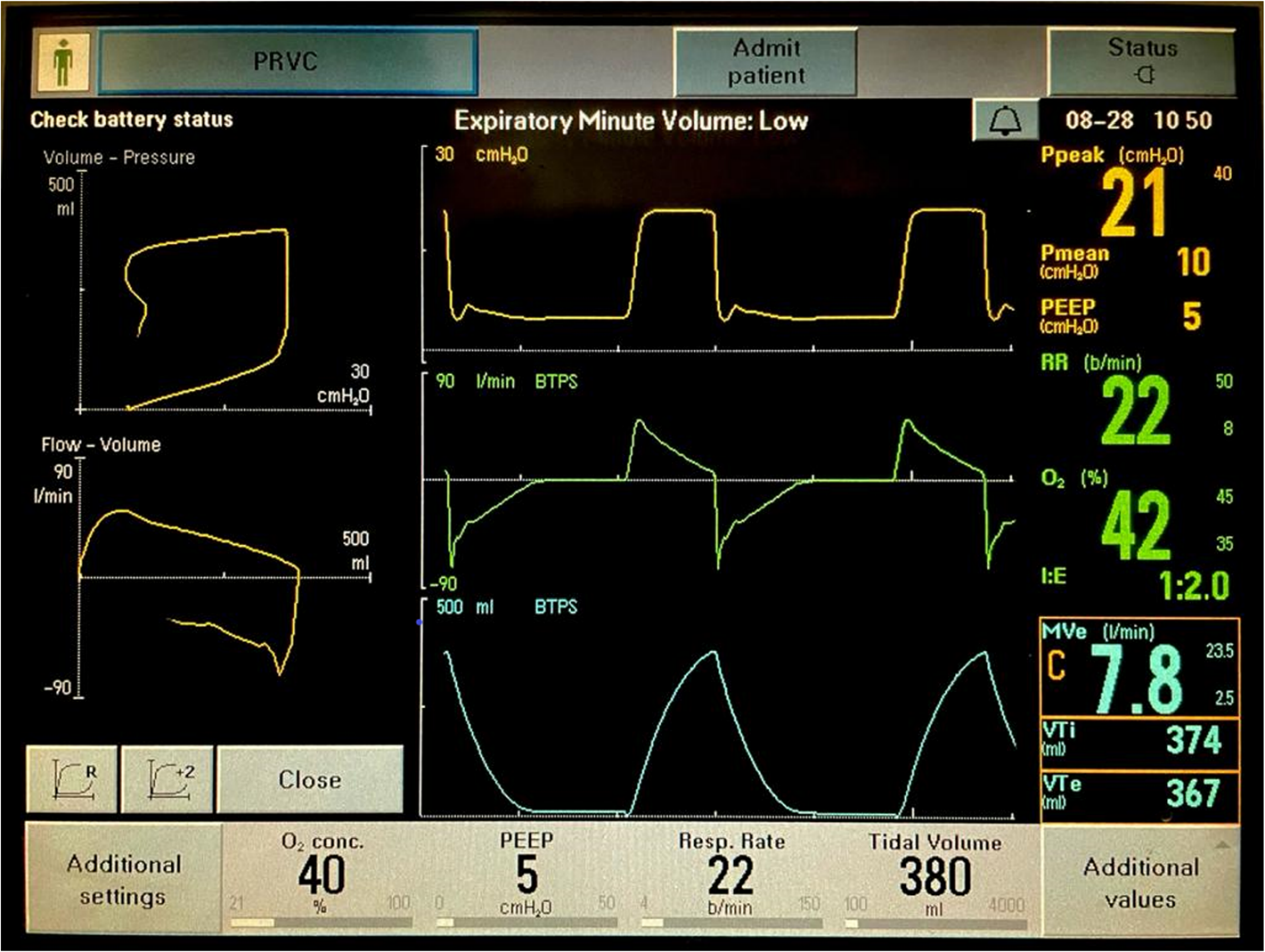
PRVC mode,  no air leak after the removal of Ryle’s tube

CXR showed that Ryle’s tube is malpositioned (Fig. 2). It was entering into the right main bronchus. Ryle’s tube’s distal end was open, and that was causing persistent leak. So, we planned to remove Ryle’s tube. After Ryle’s tube removal, the leak disappeared (Figs. 3 and 4).

## Discussion

**Fig. 5:**
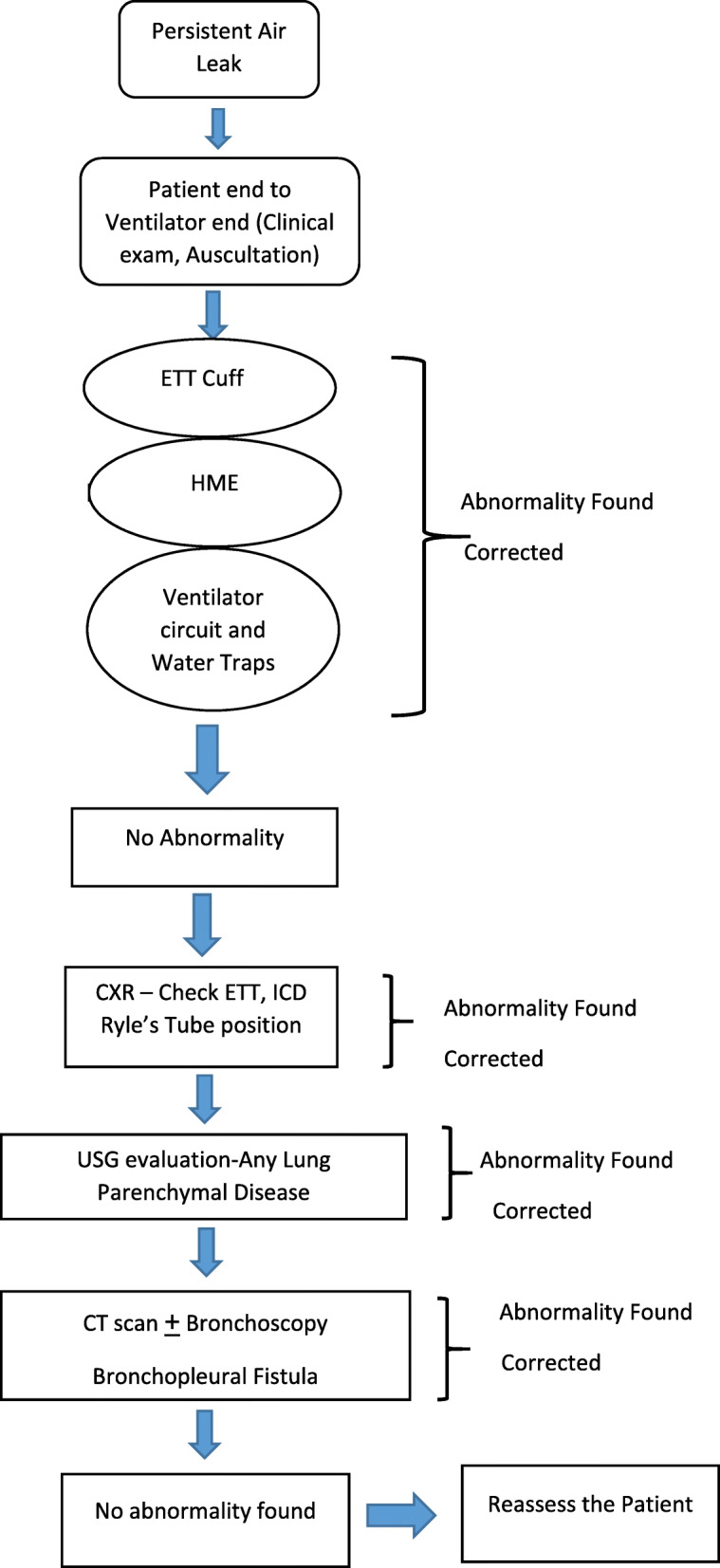
Algorithmic approach when evaluating for the cause of air leak

This case represents various important lessons. First, one should follow algorithmic approach when evaluating for the cause of air leak (Fig. 5). The assessment should begin either from the patient end or ventilator end, so that all possible causes can be easily identified. From the patient end, when we assess, we look for the endotracheal or tracheostomy cuff for cuff leak. Cuff pressure can be checked with Lange’s pressure monitor. It can become a part of routine monitoring of patients. The catheter mount which has a flip valve can be the commonest site for air leak. The HME (heat moisture exchanger) filter which has a CO_2_ sampling port, if open, can cause air leak. Then, check the ventilator circuit and water traps. The attachment of ventilator circuit to the ventilator can be loose and may get disconnected. With the help of imaging such as CXR, ultrasound, or CT scan, one can identify the anatomical and parenchymal lung diseases causing air leak. If the patient has ICD (intercostal drain), if it is not positioned properly, one of the ports can be in the subcutaneous plane and can lead to air leak. In the patient of chest trauma, the presence of open chest wound can lead to inadequate minute ventilation due to air leak.

Regarding this particular case, malpositioning of Ryle’s tube was the cause of air leak. It is not a routine practice across all the hospitals to confirm Ryle’s tube position by CXR. The confirmation by auscultation with insufflation of air method is not specific to confirm the placement of tube as the test may be false positive, and also, it may not ensure the correct positioning of Ryle’s tube [1].

In the evaluation of air leak in patients requiring mechanical ventilation as shown by Gonzalez et al., air leak can cause persistent hypercapnia, and one can take practical measures to reduce the volume of air leak [2]. McInnis et al. described, a rare case of air leak due to esophageal malignancy invading the left main bronchus [3]. Lazarus et al. described the bronchoscopic management for persistent air leak [4]. There are various causes for persistent air leak in post thoracic surgery patients. Cerfolio’s classification is used for the grading of air leaks. Dugan et al. explained various methods used in the management of such patients [5].

Identification of correct etiology is important, in order to decide about the further plan of management. In our case, it was a malpositioned Ryle’s tube which led to air leak. Doing CXR for confirmation of tubes and catheters is required. Especially, in case of Ryle's tube, as auscultation is not enough to confirm the position, CXR is considered as an optimum and feasible method [1]. The article by Fan et al. describes various methods for the confirmation of Ryle’s tube position and advantages and disadvantages of each [1].

## Conclusion

The cause of air leak in a patient described above was due to malpositioned Ryle’s tube. The physician should follow an algorithmic approach to identify the etiology correctly and manage accordingly. This case report also highlights the limitations of routine auscultation method used for confirming Ryle’s tube position.

## Data Availability

Available upon reasonable request

## References

[1] Fan EM, Tan SB, Ang SY (2017). Nasogastric tube placement confirmation: where we are and where we should be heading. Proc Singapore Healthcare..

[2] Gonzalez J, Sharshar T, Hart N, Chadda K, Raphaël JC, Lofaso F (2003). Air leaks during mechanical ventilation as a cause of persistent hypercapnia in neuromuscular disorders. Intensive Care Med..

[3] McInnis I, Walter R (2019). 1165: an unusual case of air leak in a mechanically ventilated patient. Crit Care Med.

[4] Lazarus DR, Casal RF (2017). Persistent air leaks: a review with an emphasis on bronchoscopic management. J Thoracic Dis..

[5] Dugan KC, Laxmanan B, Murgu S, Hogarth DK (2017). Management of persistent air leaks. Chest..

